# Restoring statistical validity in group analyses of motion‐corrupted MRI data

**DOI:** 10.1002/hbm.25767

**Published:** 2022-02-03

**Authors:** Antoine Lutti, Nadège Corbin, John Ashburner, Gabriel Ziegler, Bogdan Draganski, Christophe Phillips, Ferath Kherif, Martina F. Callaghan, Giulia Di Domenicantonio

**Affiliations:** ^1^ Laboratory for Research in Neuroimaging, Department of Clinical Neurosciences Lausanne University Hospital and University of Lausanne Lausanne Switzerland; ^2^ Centre de Résonance Magnétique des Systèmes Biologiques UMR5536, CNRS/University Bordeaux Bordeaux France; ^3^ Wellcome Centre for Human Neuroimaging, UCL Queen Square Institute of Neurology University College London London UK; ^4^ Institute for Cognitive Neurology and Dementia Research University of Magdeburg Germany; ^5^ Neurology Department Max Planck Institute for Human Cognitive and Brain Sciences Leipzig Germany; ^6^ GIGA Cyclotron Research Centre – in vivo imaging, GIGA Institute University of Liège Liège Belgium

**Keywords:** heteroscedasticity, motion artefact, quality control, quantitative MRI, statistical image analysis

## Abstract

Motion during the acquisition of magnetic resonance imaging (MRI) data degrades image quality, hindering our capacity to characterise disease in patient populations. Quality control procedures allow the exclusion of the most affected images from analysis. However, the criterion for exclusion is difficult to determine objectively and exclusion can lead to a suboptimal compromise between image quality and sample size. We provide an alternative, data‐driven solution that assigns weights to each image, computed from an index of image quality using restricted maximum likelihood. We illustrate this method through the analysis of quantitative MRI data. The proposed method restores the validity of statistical tests, and performs near optimally in all brain regions, despite local effects of head motion. This method is amenable to the analysis of a broad type of MRI data and can accommodate any measure of image quality.

## Graphical abstract

Motion during the acquisition of magnetic resonance imaging (MRI) data degrades image quality. We provide a data‐driven solution that assigns weights to each image of an analysis, computed from an index of image quality using restricted maximum likelihood. The proposed method restores the validity of statistical tests, is amenable to the analysis of a broad type of MRI data and can accommodate any measure of image quality.

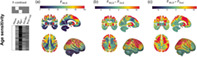

## 1 INTRODUCTION

Movement notoriously degrades magnetic resonance imaging (MRI) data, leading to prolonged examinations and increased costs in clinical applications (Andre et al., 2015; Makowski, Lepage, & Evans, 2019). Head movement also impacts the estimates of brain features extracted from MRI data (Epstein et al., 2007; Power, Barnes, Snyder, Schlaggar, & Petersen, 2012; Satterthwaite et al., 2012; Tisdall et al., 2016; Van Dijk, Sabuncu, & Buckner, 2012), and can lead to spurious detection or suppression of anatomy differences in neuroscience studies (Makowski et al., 2019). This issue is particularly acute for the study of non‐compliant patient populations, where the effects of brain disease and head motion cannot be separated (Callicott et al., 1998; Havsteen et al., 2017). Quality control procedures exist that help mitigate the effect of image degradation on analysis results. These procedures require an assessment of data quality, provided by a motion degradation index (MDI) (Castella et al., 2018; Esteban et al., 2017; Mortamet et al., 2009; Reuter et al., 2015; Rosen et al., 2018; Savalia et al., 2017). MDIs computed using dedicated image analysis routines require little labour investment and have become instrumental in the oversight of the large data cohorts that have emerged in recent years (Miller et al., 2016; German National Cohort (GNC) Consortium, 2014; Breteler, Stöcker, Pracht, Brenner, & Stirnberg, 2014; Satterthwaite et al., 2014; Glasser et al., 2016). Recent findings suggest that they may provide higher sensitivity for detecting motion artefacts than visual assessment (Alexander‐Bloch et al., 2016; Reuter et al., 2015) and, combined with supervised learning methods (Alfaro‐Almagro et al., 2018; Esteban et al., 2017; Pizarro et al., 2016), might allow the automated identification of images usable in subsequent analyses.

Quality control is typically followed by dichotomising the data into images that are either suitable (‘accept’) or unsuitable (‘exclude’) for analysis. Here, the threshold value of the MDI between the ‘exclude’ and ‘accept’ categories can be difficult to determine. Also, the effects of motion on MRI data are continuous (Alexander‐Bloch et al., 2016; Reuter et al., 2015) and it is likely that no hard categorisation of the data might achieve optimal compromise between image quality and sample size. We propose an alternative method that assigns a weight to each image within a cohort, computed from its MDI value using the restricted maximum likelihood (REML) algorithm (Diedrichsen & Shadmehr, 2005; Friston et al., 2002). The weights are specific to each image and down‐weight low quality images in subsequent analyses. We illustrate this method through the analysis of a large cohort (1,432 participants) of quantitative MRI (qMRI) data. qMRI data are in vivo biomarkers of brain microstructure (Fukunaga et al., 2010; Langkammer et al., 2010; Lutti, Dick, Sereno, & Weiskopf, 2014) that have great potential for clinical neuroscience (Barbosa et al., 2015; Khalil et al., 2011; Ropele et al., 2011). For the estimation of the weights by REML, we choose the MDI introduced by Castella et al. (2018), because this index was validated empirically against the history of head motion during data acquisition.

We show that in conventional analyses, degradation of image quality due to motion invalidates any assumption of identical variance for all samples (‘homoscedasticity’). While heteroscedasticity has little impact on the model coefficient estimates in a general linear model, the *SE* of the coefficients can be poorly estimated, leading to invalid statistical inference (Hayes & Cai, 2007). The proposed method, called QUIQI for ‘analysis of QUantitative Imaging data using a Quality Index’, restores homoscedasticity, ensuring the validity of statistical tests. With QUIQI, the improvements in homoscedasticity are superior to those obtained by inserting the MDI in the design matrix of the analysis. This global approach provides near optimal results in whole‐brain analysis of neuroimaging data, despite local effects of motion. The framework has been implemented in the popular, open‐source neuroimaging analysis software Statistical Parametric Mapping (www.fil.ion.ucl.ac.uk/spm, Wellcome Centre for Human Neuroimaging). The framework is flexible and amenable to other MDIs and to the analysis of other types of MRI data.

## 2 METHODS

### 2.1 Participant cohort

MRI data were acquired on 1,432 healthy research participants (743 females), as part of ‘BrainLaus’ (https://www.colaus-psycolaus.ch/professionals/brainlaus/) (Lorio et al., 2016; Melie‐Garcia et al., 2018; Trofimova et al., 2021), a nested project of the PsyCoLaus/CoLaus study (Firmann et al., 2008; Preisig et al., 2009). The BrainLaus study received approval from the local Ethics Committee and all participants gave their written informed consent prior to participation. The distribution of the motion degradation index values and participants' ages are shown in Figure S1. The dependence of the index on the participants' age is also shown there.

### 2.2 MRI acquisition

The MRI protocol consisted of three multi‐echo 3D fast low angle shot (FLASH) acquisitions with magnetization transfer (MTw), proton density (PDw) and T1 (T1w)‐weighted contrasts, respectively. The repetition time and nominal flip angle were 24.5 ms/6°, 24.5 ms/6° and 24.5 ms/21°, respectively. The MTw contrast was achieved with a Gaussian‐shaped RF pulse prior to the excitation (4 ms duration, 220° nominal flip angle, 2 kHz frequency offset from water resonance). 6/8/8 echo images were acquired for the MTw/PDw/T1w contrasts with a minimal echo time of 2.34 ms and an inter‐echo spacing of 2.34 ms. The image resolution was 1 mm^3^ isotropic, the field of view was 256 × 240 × 176. Parallel imaging was used along the phase‐encoding direction (acceleration factor 2; GRAPPA reconstruction (Griswold et al., 2002)). Partial Fourier was used in the partition direction with acceleration factor 6/8. The acquisition time was 7 min per contrast.

### 2.3 qMRI map computation and pre‐processing

Quantitative MRI maps were computed from the raw MRI data using the hMRI toolbox (Tabelow et al., 2019) and bespoke analysis scripts written in MATLAB (The MathWorks Inc., Natick, MA, USA). Maps of the MRI parameter R2* were computed separately from the raw echo images with MTw, PDw and T1w contrast, from the regression of the log signal with the corresponding echo times (Weiskopf, Callaghan, Josephs, Lutti, & Mohammadi, 2014). The value of the MDI described in Castella et al. (2018) was computed for each R2* map, as provided by the hMRI toolbox (Tabelow et al., 2019). Maps of Magnetization Transfer estimates (MRI parameter MTsat), were computed as described in (Helms, Dathe, & Dechent, 2008; Helms, Dathe, Kallenberg, & Dechent, 2008; Lutti et al., 2012; Lutti, Hutton, Finsterbusch, Helms, & Weiskopf, 2010; Weiskopf et al., 2013), following averaging of the echo images for each contrast to increase the signal‐to‐noise ratio (Helms & Dechent, 2009). The MTsat maps were only used for spatial normalisation of the data into a common group space (see below) and were not used in subsequent analyses.

Data were analysed using Statistical Parametric Mapping (SPM12, Wellcome Centre for Human Neuroimaging, London). The MT maps were segmented into maps of grey and white matter probabilities using Unified Segmentation (Ashburner & Friston, 2005). The nonlinear diffeomorphic algorithm Dartel (Ashburner, 2007) was used for inter‐subject registration of the tissue classes. The tissue probability maps were normalised to the stereotactic space of the Montreal Neurological Institute (MNI) template using the resulting Dartel template and the deformation fields. As described in Draganski et al.(2011), the quantitative maps were normalised using the same deformation fields but without modulation by the Jacobian determinants. Instead, a combined probability weighting and Gaussian smoothing procedure was used with a 6 mm FWHM isotropic smoothing kernel to produce tissue‐specific parameter maps while preserving the quantitative estimates.

### 2.4 Computation of image‐specific weights for data analysis

**FIGURE 1:**
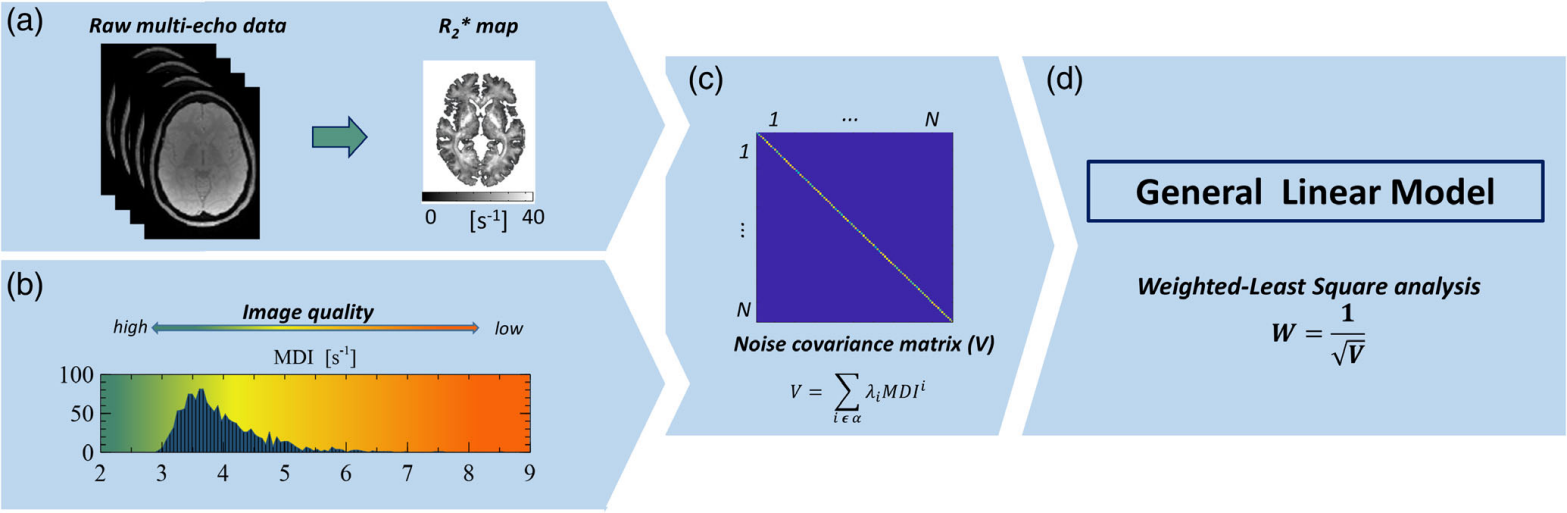
QUIQI integrates correction of motion degradation into the analysis of MRI data. For the current application, analysis data are quantitative maps of the MRI parameter R2* (a). QUIQI requires a value of the MDI for each data set of the analysis. Here, we show the distribution of the MDI values across the images used for analysis (*N* = 1,432) (b). With QUIQI, basis functions are computed from powers of the MDI and inserted into REML (Friston et al., 2002) for the computation of the noise covariance matrix *V* (c). The set of powers of the MDI, α, is pre‐defined by the user. From *V*, weights are computed that are used in the general linear model for data analysis (d)

QUIQI is integrated in the analysis of the MRI data. The first step in the estimation of the weights is specific to each application, and involves the data to be analysed and the MDI used. Here, we illustrate QUIQI on the group‐level analysis of quantitative maps of the MRI parameter R2*, computed by fitting a mono‐exponential decay model to the raw multi‐echo data (Figure 1a). For the current application, we use the MDI described in Castella et al. (2018), computed from these maps as the standard deviation of the R2* values in white matter (Figure 1b). Our choice is motivated by the empirical validation of this index, in the original study, against measures of head motion obtained from an external tracking device (Castella et al., 2018).

**FIGURE 2:**
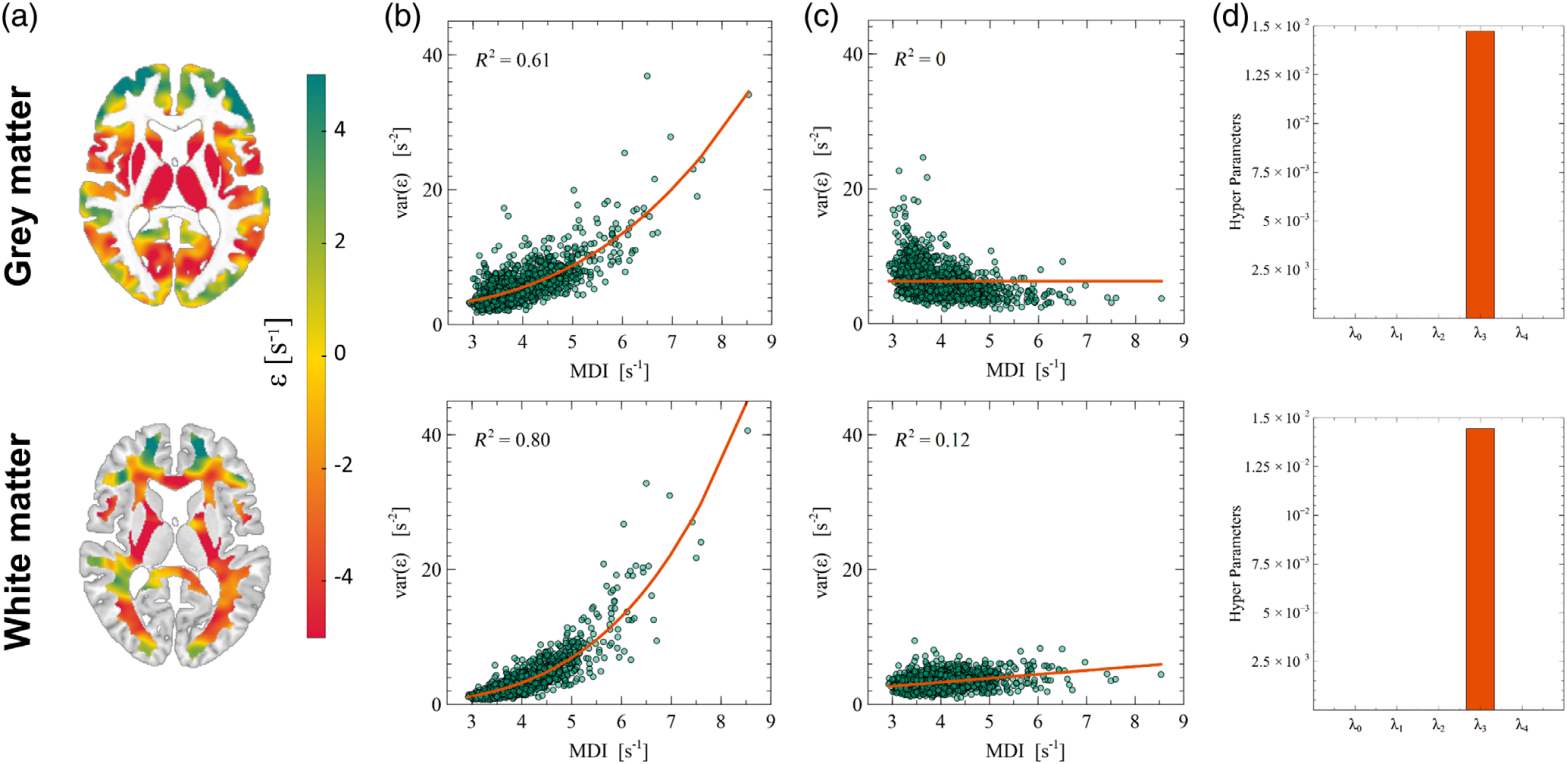
QUIQI restores homoscedasticity of the residual noise distribution in an analysis. Following model fitting, maps of the residuals are computed for each individual image (example shown in a). An estimate of image noise is computed from the variance across these residual maps and plotted against the MDI for OLS (b) and WLS analyses (c). Enforcing positivity for the hyper‐parameters (*λ*_i_ > 0) leads to a dominant cubic power in the modelling of image noise by REML (d)

With QUIQI, the values of the MDI—specific to each participant—are inserted into the REML algorithm using basis functions, that is, matrices computed from the MDI. For effective use of REML, these basis functions must capture the increased image noise due to head motion and their computation from the MDI requires a suitable mathematical form. We infer this mathematical form from the dependence of image noise on the MDI values in a standard ordinary least squares (OLS) analysis. From the polynomial dependence shown in Figure 2b, the basis functions are specified as comprising of multiple powers of the MDI, placed along the diagonal.

The following step in the calculation of the weights is independent of the application. REML estimates a set of hyperparameters **λ** that act as weights in the linear combination of the basis functions for the computation of the noise covariance matrix **V**. The matrix **V** captures the noise level in each image and is diagonal because noise is uncorrelated between participants (Figure 1c). REML estimates the **λ** hyperparameters that encode the matrix **V** by maximising the evidence lower bound (ELBO) objective function, or negative variational free energy. The idea underlying REML is that it estimates the variance–covariance of the residuals from fitting a linear model in a way that accounts for the uncertainty on the estimated model parameters (Harville, 1977). Model fitting involves jointly estimating the hyperparameters (**λ**) and voxel‐specific parameters (**β**_n_). For a design matrix **X** and image data **Y** (with *N* voxels included in the analysis) the ELBO is computed as (ignoring a constant term):

$$E\left(\lambda \right)=-\frac{N}{2}\ln\left|V_{\lambda }\right|-\frac{N}{2}\ln\left|X^{T}V_{\lambda }X\right|-\frac{1}{2}\sum_{n=1}^{N}{\left(y_{n}-X{\hat{\beta }}_{n}\right)}^{T}V_{\lambda }^{-1}\left(y_{n}-X{\hat{\beta }}_{n}\right).$$

This effectively leads to a weighted least‐square analysis (WLS) with weights $W=V^{-\frac{1}{2}}$ (Figure 1d). To compare noise models computed by REML from different sets of basis functions, we used the ELBO as a model selection criterion. The ELBO favours reducing residual errors while also penalising model complexity. The ELBO estimates were obtained from the implementation of REML within the SPM software (Friston et al., 2002).

### 2.5 Image analysis

Because the focus of this study was on the methodology involved in incorporating an MDI index into the analysis of MRI data, we restricted our analysis to R2* maps—and MDI values—computed from a single set of raw echo images per individual. We primarily focused on the analysis of the changes in R2* associated with healthy ageing, driven by changes in iron and myelin concentration in grey and white matter, respectively (Callaghan et al., 2014; Draganski et al., 2011; Yeatman, Wandell, & Mezer, 2014). Statistical analyses were carried out after estimating the parameters of a general linear model with SPM12. We included four regressors in the model, including age and the squared values of age (*age* ^*2*^), as well as gender and brain volume as variables of no interest. Analyses were conducted using the common approach of assuming identical noise levels in all quantitative maps (*Ordinary Least Squares, OLS*) as well as assuming different noise levels for each map, computed from the MDI values (*Weighted Least Squares, WLS*).

We computed measures of noise heteroscedasticity at the global level of a tissue type as well as at the individual voxel level. At the global level, our measure of heteroscedasticity was the coefficient of determination R^2^, that is, the fraction of the variance of image noise that follows a polynomial dependence on the MDI. To test for residual heteroscedasticity at the voxel level, we conducted Engle’s ARCH tests of the serial dependence of the residuals in each voxel of the MRI data, with a maximum lag of 40 data points (Engle, 1982). The fraction of voxels with significant heteroscedasticity was calculated after FDR‐correction using the Benjamini–Hochberg procedure (Glickman et al., 2014).

#### 2.5.1 Age‐associated differences in R2*

To asses the effect of QUIQI on the detection of brain‐related differences in neuroscience, we conducted statistical *F* tests of the significance of age‐related differences in R2*. We conducted these analyses, both at the global level of a whole tissue type (grey and white matter) and at the local level of a grey matter region, to assess the performance of WLS analyses in correcting local effects of head motion. The regional analyses were conducted using explicit masks defined from the grey matter maximum probability tissue labels derived from the ‘MICCAI 2012 Grand Challenge and Workshop on Multi‐Atlas Labeling’ (https://masi.vuse.vanderbilt.edu/workshop2012/index.php/Challenge_Details), computed from MRI scans originating from the OASIS project (http://www.oasis-brains.org/) and labelled data provided by Neuromorphometrics, Inc. (http://neuromorphometrics.com/) under academic subscription. The regional masks included voxels from both hemispheres.

#### 2.5.2 Specificity

We assessed the specificity of the OLS and WLS methods by monitoring the rate of false positives in two types of image analysis frequently conducted in neuroscience studies. I. In a subset of data (*N* = 123; up to 10 images per age bin of 5 years when available), the participants' age was randomly scrambled between the images before conducting the analysis of age‐associated differences in R2* described above. Any positive result would therefore be a false positive. II. In the subset of data with a narrow age range (56–58 y.o.; *N* = 129), we conducted two‐sample T tests for the comparison of two subgroups. Similarly, any positive results would therefore be a false positive. Analyses were repeated with *N*_1_ = 2, 5, 10, 20 30 and 60 images in the first group. We repeated both types of specificity analyses 1,000 times, monitoring the rate of false positives across repetitions at the voxel and cluster levels (*p* < 0.05, FWE‐corrected). For cluster‐level inference, the cluster forming threshold was *p* < 0.001 uncorrected.

#### 2.5.3 Modelling motion‐related variance in the analysis design

As a potential alternative to QUIQI, we modelled motion‐related variance in the data by inserting dedicated regressors in the design matrix (Carroll, 1982). On the model of the REML basis functions (see Section 3), these regressors contained the first to fourth powers of the MDI values. For a subset of the data within a narrow age range (56–58 y.o.; *N* = 129), we conducted statistical *F* tests of the variance of the R2* maps associated with these regressors.

## 3 RESULTS

### 3.1 The motion degradation index is a predictor of residual noise

**FIGURE 3:**
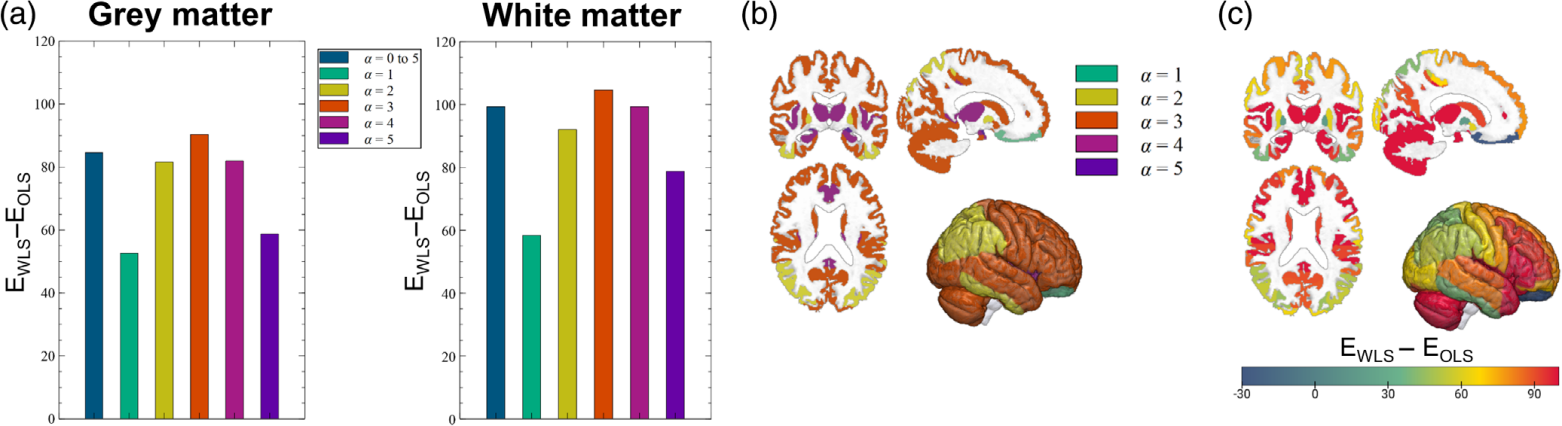
Global and local analysis of the REML ELBO. At the global level of a whole tissue type (grey or white matter), the gain in ELBO compared to OLS analyses is maximal with a single basis function in the REML estimation that contains the cubic power of the MDI values (*α* = 3, a). In grey matter regional analyses, the global optimal model (MDI^3^) was also the local optimal within most regions, primarily located in frontal areas (b). In posterior regions, the local optimal model involved the square power of the MDI. With the global optimal model, the local gain in ELBO showed a gradient in the anteroposterior direction, with the highest gain in frontal areas (c). This is consistent with typical motion of study participants during MRI examinations

The use of REML relies on the empirical observation that in a conventional analysis (*Ordinary Least Squares, OLS*), noise in MRI data can be accurately modelled from the MDI (Diedrichsen & Shadmehr, 2005). We computed an estimate of image noise as the spatial variance of the residual maps in each sample image (*var[ε]*). Figure 2a shows an example of such residual maps for grey and white matter. In an OLS analysis, the polynomial dependence of residual noise on the MDI (*R* ^2^ ~ 0.6–0.8) highlights heteroscedasticity in the data, which leads to misestimation of the precision of parameter estimates and undermines the validity of statistical tests (Figure 2b). Motivated by this dependence, QUIQI uses powers of the MDI as basis functions for estimating the noise covariance matrix. The resulting residual noise is independent of the MDI (*R* ^2^ ~ 0–0.2), restoring the homoscedasticity of the data (Figure 2c). The spread in residual noise level, estimated at the global level of a tissue type, is higher for grey matter than white matter due to partial volume effects at the tissue interface and inhomogeneities of the magnetic field. The REML hyper‐parameter estimates (λ_i_), obtained with QUIQI using a positivity constraint, shows that for the current application, the basis function that includes the cubic power of the MDI (i.e., MDI^3^) conveys the dominant contribution to the noise covariance matrix, consistently for grey and white matter (Figure 2d). This result suggests that this basis function alone might be sufficient to model accurately the effect of head motion on image noise. We will further investigate this in a subsequent step by examination of the REML ELBO (Figure 3).

We conducted Engle’s ARCH tests of residual heteroscedasticity in each voxel of the MRI data. In grey matter, the null hypothesis of no ARCH effects could be rejected in 88% and 3% of voxels for OLS and WLS, respectively (*p* < 0.05, FDR‐corrected using the Benjamini–Hochberg procedure (Glickman et al., 2014); see Figure S2). For the WLS analysis, these voxels were mainly located in sub‐cortical regions, regions affected by magnetic field inhomogeneities, or at the interface of brain tissue with its surrounding. In white matter, the null hypothesis could be rejected in 92% and 1% of voxels for OLS and WLS, respectively (*p* < 0.05, FDR‐corrected using the Benjamini–Hochberg procedure). Incidentally, WLS also reduced the number of voxels where the hypothesis of standard normally distributed noise could be rejected (Kolmogorov–Smirnov tests).

### 3.2 Optimal modelling of noise from the MDI

The evidence lower bound (ELBO), or negative variational free energy, is a model selection criterion that favours reducing residual errors while also penalising model complexity. The optimal noise model computed from the MDI maximised the ELBO provided by REML. At the global level of a given tissue type, that is, grey or white matter, a gain in ELBO of up to two orders of magnitude was obtained with WLS compared to OLS analyses (Figure 3a). Consistent with Figure 2d, the maximum gain was obtained using the MDI cubed (i.e., MDI^3^) as a basis function in the REML estimation (*global optimal model*). Including additional powers of the MDI did not increase the ELBO.

QUIQI is primarily intended for the analysis of entire images. However, for the purpose of assessing QUIQI’s ability to correct for local degradation of image quality due to head motion, we repeated the analysis separately for each region of a grey matter atlas. The global optimal model (i.e., MDI^3^) led to the highest gain in ELBO in 68% of regions. In the remaining regions, the ELBO from the global optimal model was smaller than its local counterpart by an average of 5.2. Although substantial in terms of model evidence, these differences are small compared to the gain over OLS analyses. Regions where the global and local optimal models were identical were located primarily in frontal areas, while posterior areas tended to exhibit locally optimal models with a lower power of the MDI (Figure 3b). This anterior–posterior gradient is also apparent in the increase in ELBO compared to OLS analyses: the highest gains are observed in frontal regions (Figure 3c).

The global optimal model for the current application, with only MDI^3^ as a basis function in the REML estimation, was used in all subsequent analyses conducted with QUIQI.

### 3.3 QUIQI increases analysis sensitivity to brain differences

**FIGURE 4:**
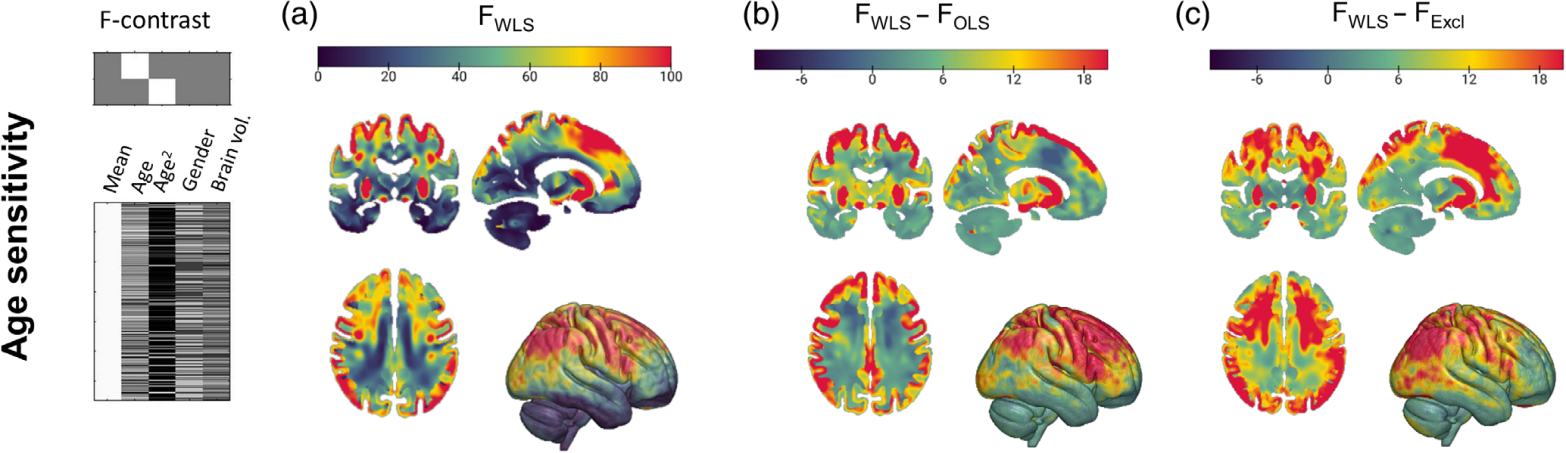
QUIQI increases the sensitivity of MRI data analysis. The effect of QUIQI on the analysis of brain‐related differences was assessed using statistical *F* tests of the dependence of the MRI data with age (a). Compared with OLS analyses, QUIQI leads to region‐specific decreases or increases in F‐scores due to the restored noise homoscedasticity (b). Exclusion of the 30% most degraded images was required to restore noise homoscedasticity in OLS analyses. WLS analyses yield higher age‐sensitivity over the whole brain (c)

Figure 4a shows a map of statistical F‐scores of the dependence of the MRI data on age, obtained using QUIQI with the noise model optimal for the current application. As previously reported (Callaghan et al., 2014; Draganski et al., 2011), the most prominent age‐related differences in R2* were located in sub‐cortical grey matter due to a local increase in iron concentration with age and in frontal white matter due to a peak in axonal myelination around midlife (Slater et al., 2019).

Compared with OLS analyses, QUIQI leads to region‐specific decreases or increases in F‐scores (Figure 4b) but because QUIQI restores noise homoscedasticity, WLS analyses are more sensitive to true age effects. We compared the age sensitivity of WLS analyses with that of OLS analyses after exclusion of the fraction of the images with the highest MDI values (i.e., the most degraded images, see Figure S3a). From the multiple fractions considered (3, 7, 13, 20 and 30% (Esteban et al., 2017; Mortamet et al., 2009; Pizarro et al., 2016; Rosen et al., 2018)), we selected the one that led to similar noise homoscedasticity to WLS analyses. In grey matter, this was achieved after removing 30% of the images (*R* ^2^ = 0.11, see Figure S3b). With this fraction of excluded data, heteroscedasticity was still present in white matter (*R* ^2^ = 0.43) but higher values were deemed too prohibitive to be considered. WLS analyses led to higher age‐sensitivity than exclusion OLS analyses in both tissue types (Figure 4c).

### 3.4 QUIQI preserves the specificity of statistical analyses

Line 1 of Table 1 shows the rate of false positives, obtained from statistical *F* tests of age‐related differences after scrambling of the age regressor (analysis I). The rate of false positives was within the expected range at the voxel and cluster levels, for both OLS and WLS analyses (*p* < 0.05, FWE‐corrected). Line 2 of Table 1 shows the rate of false positives in group comparisons (analysis II). The false positive rate increased similarly for OLS and WLS analyses in unbalanced group comparisons (Salmond et al., 2002). For white matter, a false positive rate within 0.05 was found for cluster‐level inference with *N*_1_ ≈ 5 or more images in the first group. More false positives were observed for grey matter. The false positives were primarily located in cortical regions affected by magnetic field inhomogeneities (e.g., orbitofrontal cortex and temporal lobes, see Figure S4).

**TABLE 1:** Specificity of MRI analyses: false positive rates in one‐sample and two‐sample analyses

		GM					WM				
One sample *t* test	OLS	0.02/0.022					0.029/0.023				
	WLS	0.027/0.022					0.026/0.013				
Two sample *t* tests		*N*_1_ = 2	*N*_1_ = 5	*N*_1_ = 10	*N*_1_ = 30	*N*_1_ = 60	*N*_1_ = 2	*N*_1_ = 5	*N*_1_ = 10	*N*_1_ = 30	*N*_1_ = 60
	OLS	0.860/0.245	0.509/0.104	0.203/0.055	0.074/0.049	0.051/0.042	0.618/0.182	0.254/0.066	0.132/0.043	0.076/0.021	0.072/0.032
	WLS	0.895/0.268	0.601/0.112	0.332/0.061	0.102/0.047	0.075/0.046	0.592/0.167	0.256/0.05	0.117/0.03	0.068/0.024	0.071/0.033

*Note*: The false positive rates are reported at the voxel/cluster‐levels. For one‐sample *t* tests, the false positive rate was below 0.05, the *p* value used for statistical significance, for both OLS and WLS analyses. For two‐sample *t* tests the false positive rate, reported for different numbers of subjects *N*_1_ in the first group, increased similarly for OLS and WLS analyses in unbalanced group comparisons (Salmond et al., 2002). The higher number of false positives in grey matter arises from cortical regions affected by magnetic field inhomogeneities leading to bias on the R2* estimates (see Figure S4).

### 3.5 Modelling motion‐related variance from covariates in the analysis design does not restore homoscedasticity

**FIGURE 5:**
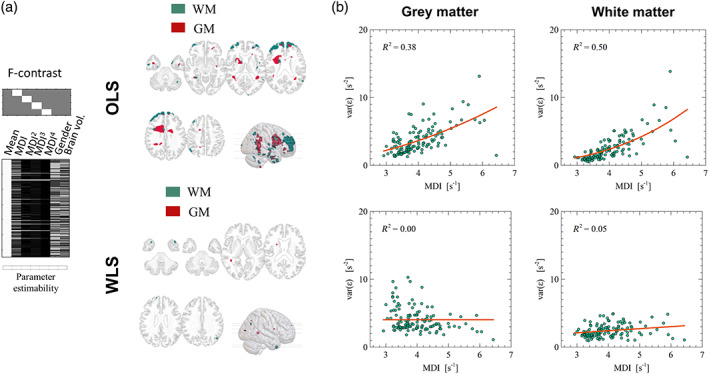
Using motion regressors as design covariates does not restore heteroscedasticity. In OLS analyses, statistical *F* tests of the variance of the R2* maps associated with powers of the MDI show statistically significant results in 3.2% and 5.5% of voxels in grey and white matter, respectively (*p* < 0.05, FWE‐corrected) (a). In WLS analyses, significant results are found in 0.1% of voxels only (*p* < 0.05, FWE‐corrected). Noise heteroscedasticity remains present for both tissue types in OLS analyses, despite the motion regressors, but is not present in WLS analyses (b)

Figure 5 shows the results of inserting a set of regressors computed from the MDI in the image analysis. For OLS analyses, statistically significant results were found in 3.2% and 5.5% of voxels in grey and white matter, respectively (*p* < 0.05, FWE‐corrected). For grey matter, these voxels were primarily located in frontal regions (see Figure 5a). For WLS analyses, statistically significant results were only found in 0.1% of voxels in grey and white matter (*p* < 0.05, FWE‐corrected). Residual analysis showed that noise heteroscedasticity remains in the data for both tissue types with OLS analyses, despite the motion regressors. Noise heteroscedasticity is not present in WLS analyses (Figure 5b).

## 4 DISCUSSION

In this study, we introduce a method that accounts for the degradation of image quality due to motion in the analysis of MRI data. This method addresses an important limitation of existing approaches for quality control, which only enable the removal of the most degraded images from analysis. From an index of image quality, the proposed method computes weights that capture the noise level in each image, leading to increased sensitivity to brain change in an analysis. This method is based on restricted maximum likelihood, available in most image analysis software suites. The implementation used here, within the SPM software (Friston, Stephan, Lund, Morcom, & Kiebel, 2005), is commonly used to account for differential noise levels in, for example, group‐comparison studies (‘*non‐sphericity’*). Here, we extend this methodology to the statistical analysis of structural MRI data, using an index of image quality to estimate the noise level in each individual image. We validated this method using a large cohort of 1,432 subjects, which allowed the design of multiple analyses to test different aspects of the method. The analysis code used in this study is publicly available (Lutti, 2021).

In conventional analysis methods, the increase of the noise level due to motion leads to a violation of the homoscedasticity assumption of statistical tests. By estimating the noise level in each image from its MDI value, QUIQI restores the validity of this assumption, both at the global level of a tissue type (i.e., grey or white matter) and in individual voxels of the MRI data. Voxels where significant heteroscedasticity remain with QUIQI are primarily located in regions where other non‐motion mechanisms may be at play: sub‐cortical areas (delineation errors), interface of brain tissue (partial volume effects) and regions of inhomogeneous magnetic field (bias of the R2* estimates). We tested an alternative method to QUIQI, based on the insertion of the MDI as a covariate in the design matrix of the analyses. However, we show that this alternative method does not successfully correct for noise heteroscedasticity.

To illustrate the effect of QUIQI on the analysis of MRI data, we conducted analyses of age‐associated brain differences. OLS analyses led to spurious statistical results due to noise heteroscedasticity in the data. Restoring noise homoscedasticity in OLS analyses required the removal of the 30% of the images most affected by motion in grey matter (‘exclusion analyses’). This ratio was even higher for white matter. The sensitivity of WLS analyses to age‐related differences was superior to that of exclusion analyses over the whole brain. The higher sensitivity of WLS analyses did not inflate the rate of false positives (Table 1).

QUIQI corrects local effects of head motion optimally—or near optimally—in whole‐brain analyses, the archetypal use of MR images for neuroscience. The increase in ELBO with QUIQI was largest in frontal brain regions and the local optimal noise model involved a higher power of the MDI there. This is consistent with the supine position of the study participants during MRI examination, with the back of the head resting on the scanner table, and provides further evidence of the ability of QUIQI to correct the effects of motion on MRI data quality.

We implemented QUIQI by enforcing positivity of the hyper‐parameter values estimated by REML (spm_reml_sc). This is by no means a requirement because allowing negative hyper‐parameter values works equally well (Figure S5). Our choice was primarily guided by the fact that enforcing positivity allowed us to identify a single basis function (MDI^3^) as sufficient to effectively model noise in the data (Figure 2d). With a single basis function, local analysis results (Figure 3b and c) reflect the ability of the method to correct for local effects of motion in a global analysis of a whole tissue type (e.g., grey or white matter). This would not be the case with several basis functions because the hyper‐parameter values, which combine the basis functions in the estimation of the noise covariance matrix, would differ in a local and global analysis. Here, we provide a version of spm_est_non_sphericity that calls spm_reml_sc to enforce positive hyperparameter estimates (Lutti, 2021).

The proposed method is amenable to most types of MRI data and motion degradation indices. However, we expect that the optimal noise model might differ depending on the type of MRI data to be analysed. This study outlines a process for the identification of the optimal noise model based on the estimation of noise heteroscedasticity (Figure 2) and on the maximisation of the REML ELBO (Figure 3). To facilitate uptake by interested users, we provide a customised version of the hMRI toolbox (Tabelow et al., 2019) with a dedicated QUIQI module available from the GUI (Lutti, Di Domenicantonio, Corbin, Phillips, & Callaghan, 2021). The powers of the MDI used to define the REML basis functions can be set freely from the user interface. QUIQI allows the plotting and polynomial fitting of image noise versus the MDI (‘QUIQI check’). On the model of this study, this allows users to assess heteroscedasticity levels in their analysis data, with or without QUIQI, and helps identify the optimal set of basis functions for the REML estimation. We emphasise that this feature runs independently from the analysis and that the polynomial fits are not used subsequently. Instead, the use of REML for the computation of the weights provides estimates of ELBO as measures of noise model efficiency (the analysis code attached to this manuscript includes a customised version of the ‘spm_est_non_sphericity’ SPM function that provides the estimate of the REML ELBO computed by SPM). Similarly, we do not recommend the computation of the weights directly from the global noise estimates as factors independent of motion degradation such as analysis model or data type (e.g., field inhomogeneities here) may then have an impact on the weight estimates.

Here, we emphasised the principles of the method and provided a detailed assessment of its performance. For illustration, we therefore chose the analysis of brain phenotype data that can be computed from one raw image type, that is, quantitative maps of the MRI parameter R2* (Lutti, Di Domenicantonio, Kherif, & Draganski, 2021). The extension of this method to the analysis of quantitative maps computed across multiple types of raw images, each with their own degree of motion degradation, is currently ongoing. Another important field of application is the analysis of differences in brain morphology (e.g., grey matter volume or cortical thickness), the most widespread phenotypical measures extracted from MR images. Such applications will highlight the effect of image processing (segmentation) on the sensitivity of analysis to motion. While QUIQI can be readily used with different MDIs, we highlight the importance of the specificity and sensitivity of the index to motion degradation (Castella et al., 2018), which drive the efficacy of the method. Potential sources of confound on image‐based MDIs (e.g., brain disease) should be closely investigated (Supporting Information, Appendix S1).

## 5 CONCLUSION

We introduce a method that accounts for the degradation of image quality due to motion in the analysis of MRI data. From an index of image quality, this method computes weights specific to each individual structural image of a statistical analysis. We show that in conventional analysis methods, the increased noise level due to motion leads to a violation of the homoscedasticity assumption of statistical tests. By estimating the noise level in each image from its MDI value, the proposed method restores the validity of this assumption. This method was compared with existing approaches for quality control based on the removal of the most degraded images from analysis. We show that the proposed method improves the compromise between image quality and sample size, leading to increased sensitivity to brain change. The improvements in homoscedasticity are also superior to those obtained by modelling motion degradation as a confounding effect in the design matrix of the analysis.

The proposed method is based on restricted maximum likelihood, available in most image analysis software suites, and can be readily used with different indices of motion degradation. The specificity and sensitivity of the index to the degradation of MRI data to motion is paramount to ensure optimal performance of the method.

## CONFLICT OF INTERESTS

The authors declare no competing interests.

## AUTHOR CONTRIBUTIONS

A.L. designed the experiment. A.L., G.D.D, F.K. and B.D. collected the data. A.L., N.C. and G.D.D. developed techniques and analysed the data. A.L. wrote the paper. A.L., N.C., J.A., G.Z., B.D., C.P., F.K. and M.F.C. designed the analysis and interpreted the results. A.L., N.C., J.A., G.Z., B.D., C.P., F.K., M.F.C. and G.D.D discussed and edited the manuscript.

## ETHICS APPROVAL STATEMENT

This study received approval from the local Ethics Committee and all participants gave their written informed consent prior to participation.

## Supporting information


**APPENDIX** S1. Supporting Information

**Figure S1** Distribution of the MDI values (a) and participants' age (b) across the datasets used for analysis (*N* = 1,432), and dependence of the MDI on participant’s age (c)

**Figure S2** QUIQI reduces residual heteroscedasticity in each voxel of the MRI data. In grey matter, significant heteroscedasticity was found in 88% and 3% of voxels for OLS and WLS respectively (*p* < 0.05, FDR‐corrected using the Benjamini–Hochberg procedure). In white matter, significant heteroscedasticity was found in 92% and 1% of voxels for OLS and WLS respectively (*p* < 0.05, FDR‐corrected using the Benjamini–Hochberg procedure)

**Figure S3** With OLS analyses, restoring homoscedasticity requires removal of 30% of the subjects or more. We assessed noise homoscedasticity after exclusion of up to 30% of the most degraded images from OLS analyses (a). We estimated noise homoscedasticity by fitting the dependence of residual noise on the MDI with a polynomial function of order 3. In grey matter, restoring noise homoscedasticity requires the removal of 30% of the images (goodness of fit: *R* ^2^ = 0.11; (b)). In white matter, noise homoscedasticity comparable to that of WLS analyses was not achieved

**Figure S4** Regions affected by magnetic field inhomogeneities drive the occurrence of false positives in unbalanced group comparisons. Spatial distribution of the voxels showing significant differences at least twice out of 1,000 repetitions in the analysis of specificity in group comparisons. The number of subjects in the first group was *N*_1_ = 5. Similarly for OLS (a) and WLS (b) analyses, these voxels are primarily located in regions affected by magnetic field inhomogeneities (e.g., temporal lobes, orbitofrontal cortex)

**Figure S5** Allowing for negative REML hyper‐parameter values leads to a similar performance of QUIQI. Allowing for negative hyper‐parameter values (*λ* > 0 or *λ* < 0), noise homoscedasticity is restored as when positive hyper‐parameters is enforced (*λ* > 0) (a). Note that consistently with the REML estimation, the polynomial fitting of the dependence of image noise on the MDI in a) also allowed for negative coefficients. The gain in ELBO compared to OLS analyses is also in a similar range (b)

## Data Availability

Material related to this paper is available here: https://zenodo.org/record/4889895#.YdgV6CDMJPY This material also includes the results of the analyses presented in this article, conducted on the available data.
