# Supplementary figures and images for: Restoring statistical validity in group analyses of motion‐corrupted MRI data
Source: Hum Brain Mapp. 2022 Feb 3;43(6):1973–83. doi: 10.1002/hbm.25767 (PMC8933245; doi:10.1002/hbm.25767)

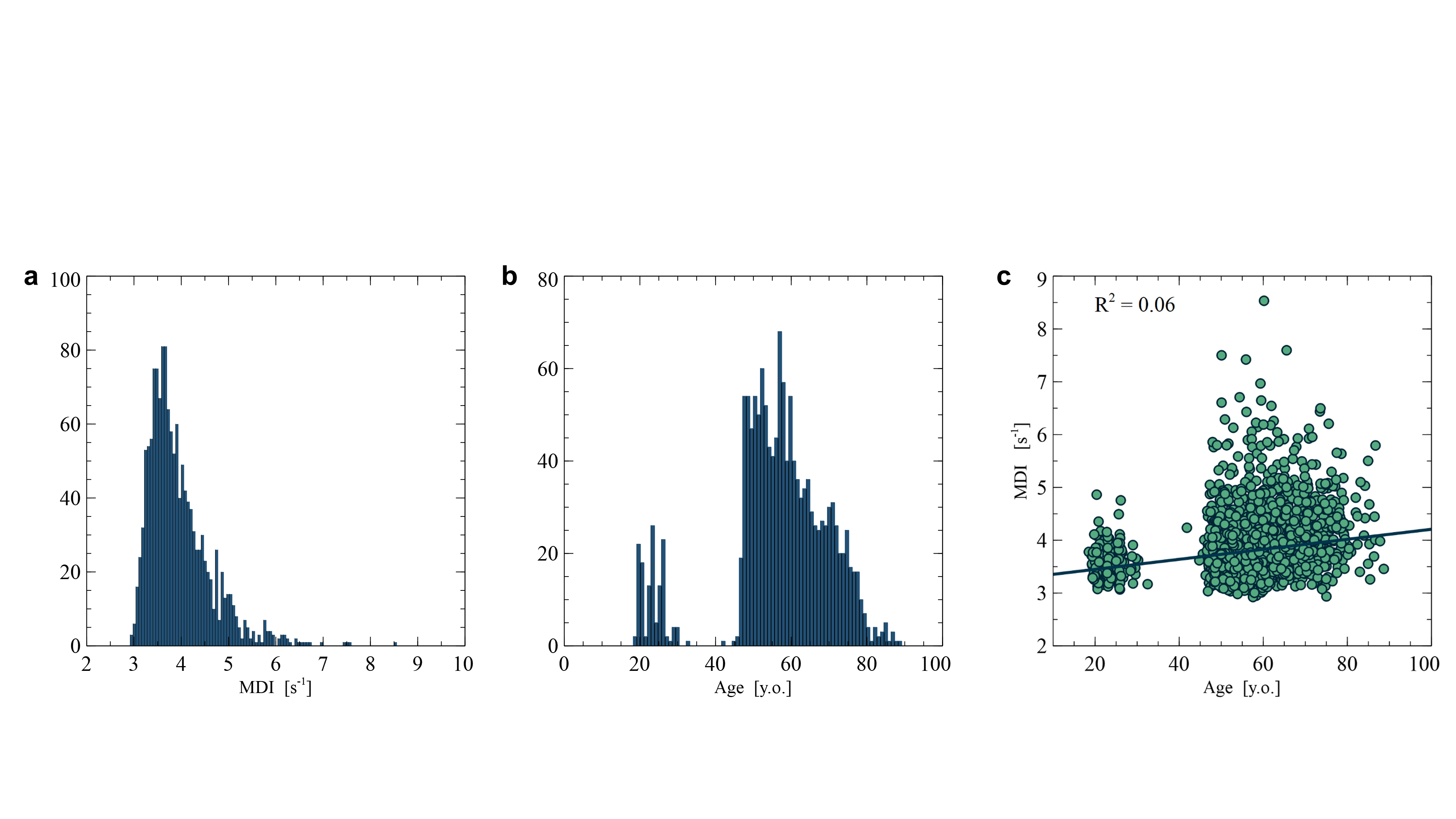

Supplement: Supplementary file 2 — Figure S1 Distribution of the MDI values (a) and participants' age (b) across the datasets used for analysis (N = 1,432), and dependence of the MDI on participant’s age (c) [file HBM-43-1973-s001.tif]

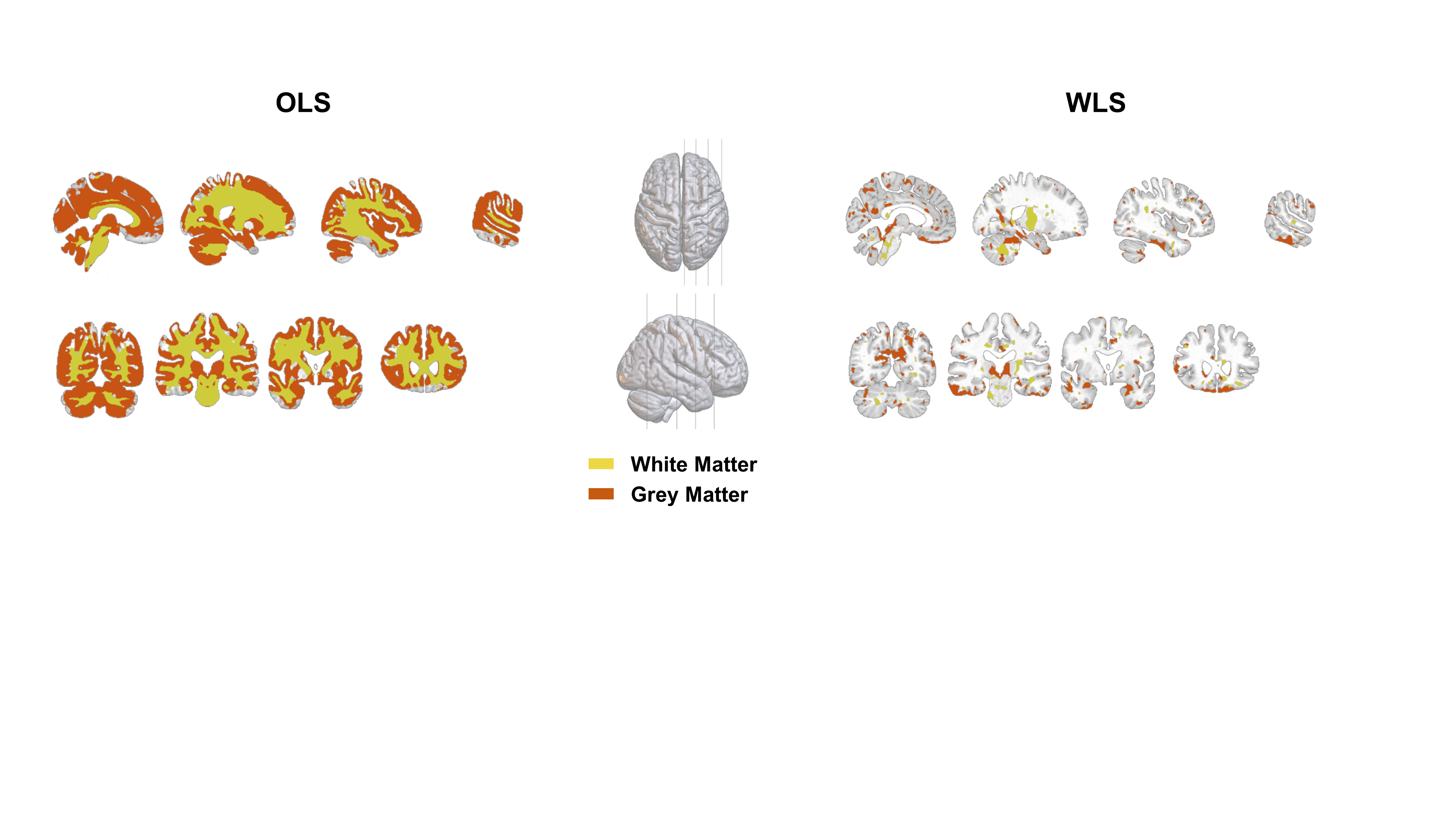

Supplement: Supplementary file 3 — Figure S2 QUIQI reduces residual heteroscedasticity in each voxel of the MRI data. In grey matter, significant heteroscedasticity was found in 88% and 3% of voxels for OLS and WLS respectively (p < 0.05, FDR‐corrected using the Benjamini–Hochberg procedure). In white matter, significant heteroscedasticity was found in 92% and 1% of voxels for OLS and WLS respectively (p < 0.05, FDR‐corrected using the Benjamini–Hochberg procedure) [file HBM-43-1973-s004.tif]

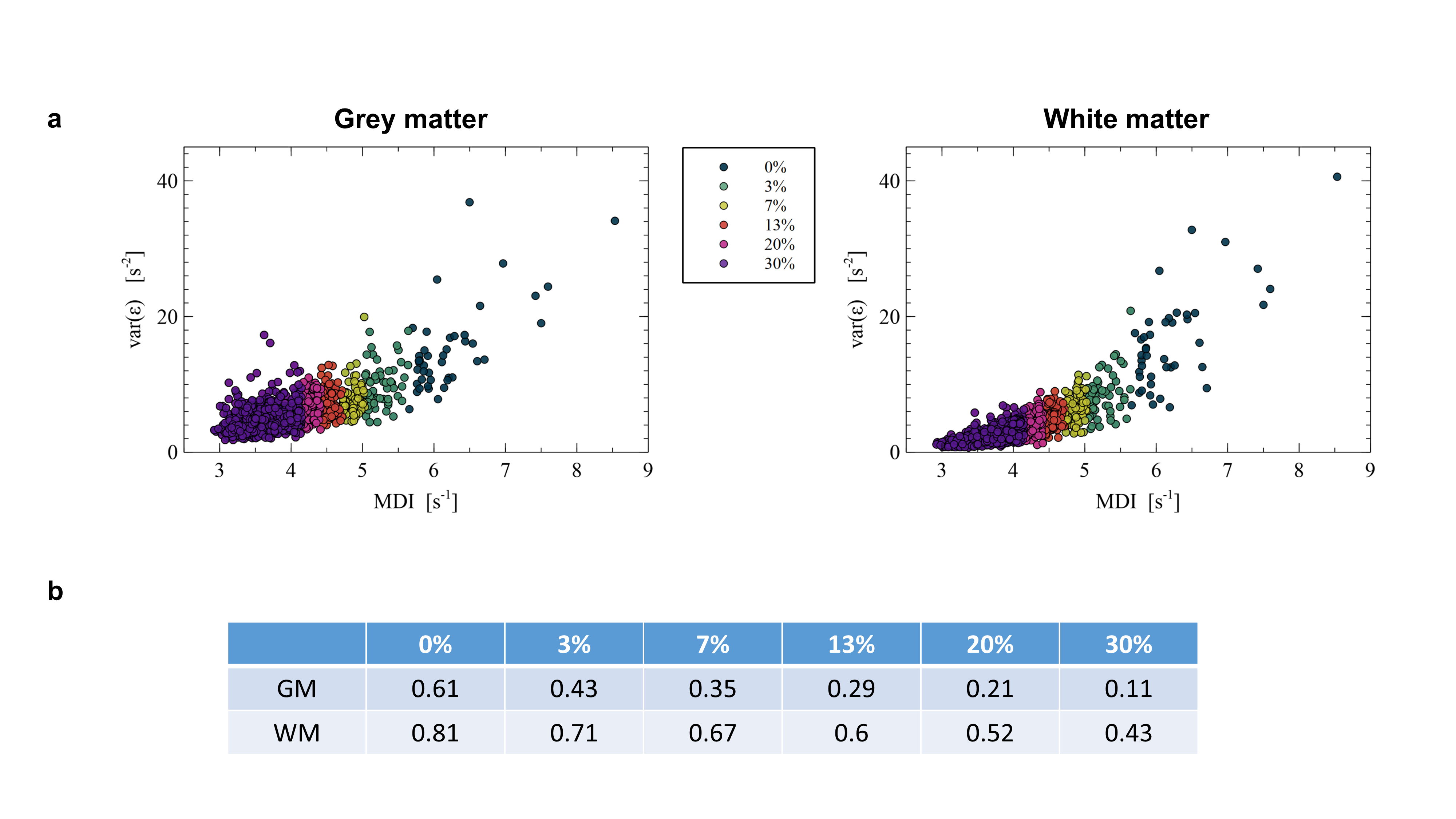

Supplement: Supplementary file 4 — Figure S3 With OLS analyses, restoring homoscedasticity requires removal of 30% of the subjects or more. We assessed noise homoscedasticity after exclusion of up to 30% of the most degraded images from OLS analyses (a). We estimated noise homoscedasticity by fitting the dependence of residual noise on the MDI with a polynomial function of order 3. In grey matter, restoring noise homoscedasticity requires the removal of 30% of the images (goodness of fit: R 2 = 0.11; (b)). In white matter, noise homoscedasticity comparable to that of WLS analyses was not achieved [file HBM-43-1973-s006.tif]

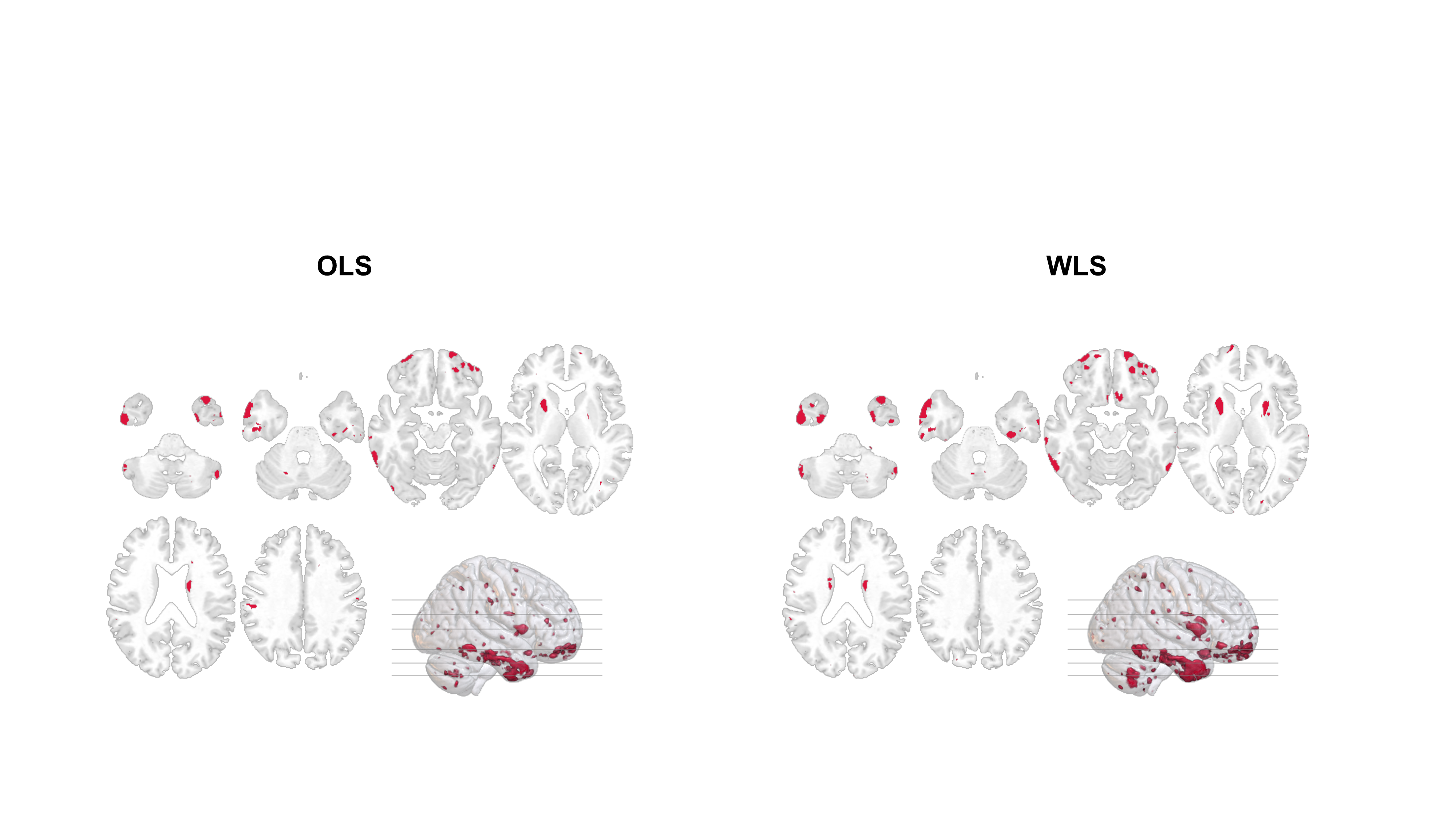

Supplement: Supplementary file 5 — Figure S4 Regions affected by magnetic field inhomogeneities drive the occurrence of false positives in unbalanced group comparisons. Spatial distribution of the voxels showing significant differences at least twice out of 1,000 repetitions in the analysis of specificity in group comparisons. The number of subjects in the first group was N 1 = 5. Similarly for OLS (a) and WLS (b) analyses, these voxels are primarily located in regions affected by magnetic field inhomogeneities (e.g., temporal lobes, orbitofrontal cortex) [file HBM-43-1973-s002.tif]

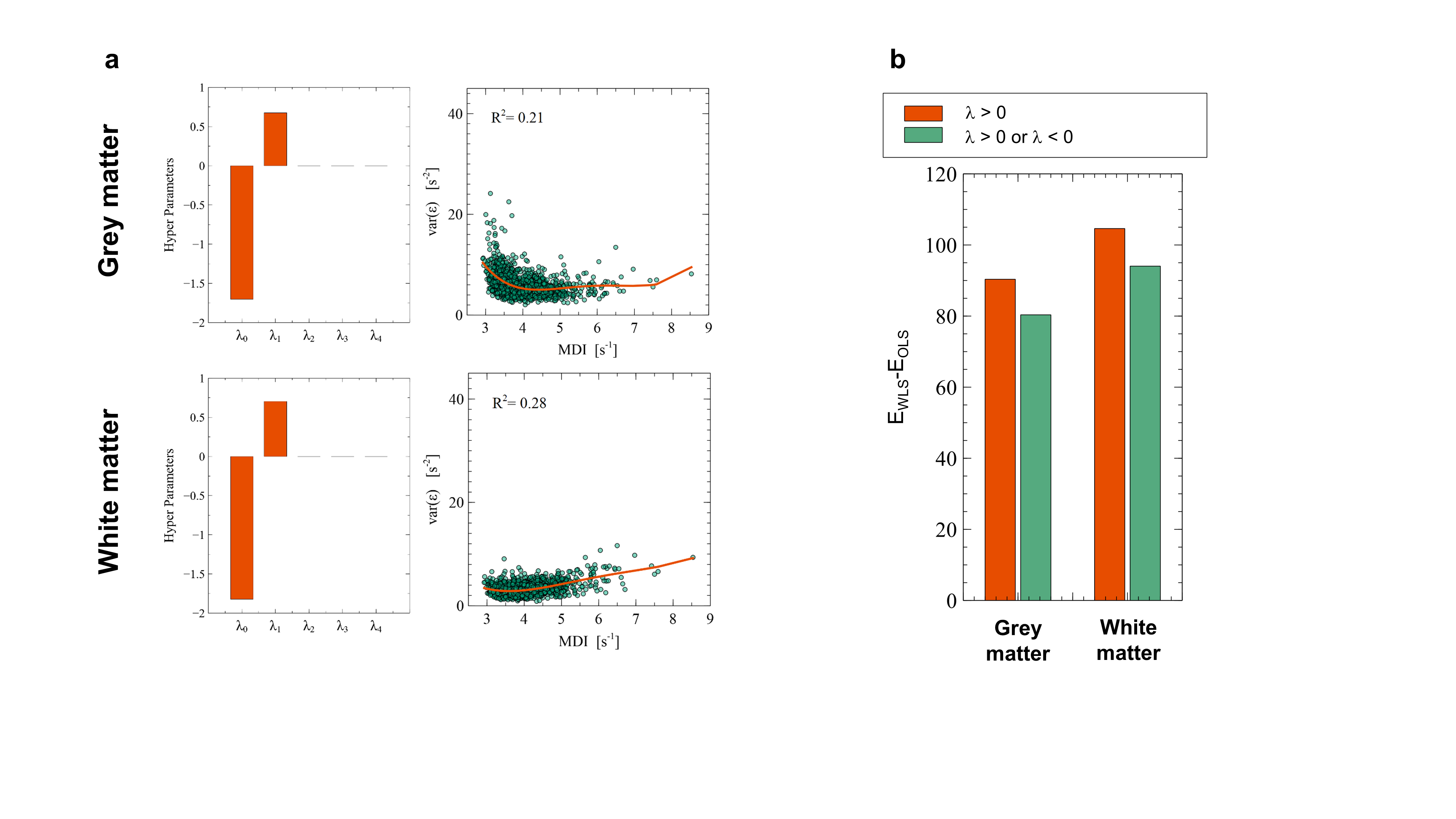

Supplement: Supplementary file 6 — Figure S5 Allowing for negative REML hyper‐parameter values leads to a similar performance of QUIQI. Allowing for negative hyper‐parameter values (λ > 0 or λ < 0), noise homoscedasticity is restored as when positive hyper‐parameters is enforced (λ > 0) (a). Note that consistently with the REML estimation, the polynomial fitting of the dependence of image noise on the MDI in a) also allowed for negative coefficients. The gain in ELBO compared to OLS analyses is also in a similar range (b) [file HBM-43-1973-s005.tif]
